# *Toxoplasma gondii* Infection in Patients with Cardiovascular Diseases from Western Romania: A Case–Control Study

**DOI:** 10.3390/life13071575

**Published:** 2023-07-17

**Authors:** Angela Dragomir, Maria Alina Lupu, Rodica Lighezan, Ana Alexandra Paduraru, Tudor Rares Olariu

**Affiliations:** 1 Discipline of Parasitology, Department of Infectious Diseases, Victor Babes University of Medicine and Pharmacy, 300041 Timisoara, Romania; angela.dragomir@umft.ro (A.D.); lighezan.rodica@umft.ro (R.L.); paduraru.ana@umft.ro (A.A.P.); 2 Clinical Laboratory, Institute of Cardiovascular Diseases, 300310 Timisoara, Romania; 3 Center for Diagnosis and Study of Parasitic Diseases, Department of Infectious Disease, Victor Babes University of Medicine and Pharmacy, 300041 Timisoara, Romania; 4 Patogen Preventia, 300124 Timisoara, Romania; 5 Regional Blood Transfusion Center, 300737 Timisoara, Romania; 6 Clinical Laboratory, Municipal Clinical Emergency Teaching Hospital, 300254 Timisoara, Romania

**Keywords:** *Toxoplasma gondii*, antibodies, seroprevalence, cardiovascular diseases, epidemiology, Romania

## Abstract

(1) Background: *Toxoplasma gondii* infects approximately one third of the world’s human population. The seroepidemiology of *T. gondii* in cardiovascular patients is poorly discussed in the existing literature. We aimed to evaluate, for the first time, the seroprevalence of *T. gondii* in cardiovascular patients from Western Romania. (2) Methods: Serologic testing to demonstrate the presence of *T. gondii* antibodies was conducted in 256 patients with cardiovascular diseases and 261 matched blood donors. (3) Results: The overall seroprevalence of *T. gondii* antibodies was 64.06% in patients with cardiovascular diseases and 52.88% in blood donors and tended to increase with age in both groups. The seroprevalence of *T. gondii* antibodies was significantly higher in cardiovascular male patients (69.94%) compared to male blood donors (55.69%) (*p* = 0.006). When compared to the control group, a significantly higher prevalence of *T. gondii* antibodies was found among patients with hypertension (82.35%; *p* = 0.01) and unstable angina (67.56%; *p* = 0.02). (4) Conclusions: This study brings new epidemiological information on the prevalence of *T. gondii* in Romanian cardiovascular patients. *T. gondii* seroprevalence was significantly higher in patients with hypertension and unstable angina, suggesting that individuals with these diagnoses may be more frequently infected with *T. gondii*. This study may be a valuable starting point for further research to better evaluate the impact of *T. gondii* exposure on patients with cardiovascular diseases.

## 1. Introduction

*Toxoplasma gondii* is an apicomplexan parasite that can infect humans and a broad range of animals and birds. Approximately one third of the world’s human population is infected with this protozoan [1,2,3]. Due to its socioeconomic and public health impacts, this opportunistic organism has reached global interest. However, despite its prevalence, toxoplasmosis was listed among the neglected parasitic infections by the Center for Disease Control and Prevention (CDC) [2,3,4]. 

All three distinct morphological stages of *T. gondii* are infective: (i) tachyzoite is the dissemination form, with rapid multiplication, present in acute infection; (ii) bradyzoite is the result of tachyzoite conversion, has slow multiplication, and with other bradyzoites forms the tissue cyst, present in chronic infection; (iii) sporozoite is present in mature (sporulated) oocysts [2,5].

The life cycle of *T. gondii* functions in a prey–predator system involving domestic and wild cats (exclusive definitive hosts which harbor sexual reproduction) and most warm-blooded mammals, birds, and humans (intermediate hosts which harbor asexual reproduction). In humans, *T. gondii* exists in either tachyzoite or bradyzoite stages [3,5]. 

Humans can become infected through different pathways: (i) ingestion of vegetables, fruits, water, clams, oysters, or mussels contaminated with sporulated oocysts (containing sporozoites); (ii) eating raw or undercooked meat/primary offal containing bradyzoites organized in tissue cysts; (iii) transplacental transmission by tachyzoites (leading to congenital toxoplasmosis) or transfusion of tachyzoite-infected blood (possible if the donor is recently infected and is parasitemic at the time of blood sampling); (iv) organ transplantation (if the organs contain tachyzoites or tissue cysts). Although not common, non-pasteurized (raw) goat’s milk may be a source of acquired infection: tachyzoites were suggested to have the ability to penetrate the mucosal tissue of the host [1,2,5,6,7,8,9]. *T. gondii* interaction with human cells (particularly epithelial cells, macrophages, neurons, and muscle cells) aims its internalization, followed by rapid multiplication and dissemination throughout the organism. Seven to ten days after infection, tissue cysts arise and may persist throughout life (predominantly in the brain, skeletal muscles, cardiac muscles, and retina) due to their long-term survival and their ability to elude immunomediated destruction [1,3,10].

Genetic studies of isolates from the United States and Europe identified three major multilocus genotypes stable in time and space, equivalent to clonal lineages: types I, II, and III. There is approximately 1–2% genetic divergence (at the DNA sequence level) between these lineages. Multichromosome and multilocus genotyping revealed more frequent genetic exchanges and genetic drift, resulting in a greater genetic diversity and a much more complex population structure. Based on sequence-based analyses, 12 haplogroups have been described (including the three initially described types I, II, and III), with highly diverse and truly atypical isolates which cannot be clustered into one of these haplogroups [5].

*T. gondii* pathogenesis spans acute, chronic, congenital, and ocular infections, and reactivated chronic toxoplasmosis (in immunocompromised patients) [2]. Clinical severity of toxoplasmosis is influenced by: (i) the immune status of the infected individual (involving both innate and adaptive immune responses); (ii) the strain of *T. gondii* (usually, genotypes with a majority of type I alleles are more virulent) [2,5,9]; (iii) the pathway of infection (oocyst-induced infections are suggested to be clinically more severe compared to tissue cyst-acquired infections) [9,11]. In most immunocompetent individuals, postnatally infection with *T. gondii* is asymptomatic. In about 10% of cases, patients can describe flu-like or mononucleosis-like symptoms [2,12]. However, in healthy individuals infected with *T. gondii,* myocarditis, pneumonitis, encephalitis, polymyositis, or hepatitis can rarely arise [12]. The risk of maternal–fetal transmission increases with gestational age, and fetal involvement is most severe when the mother acquires the infection early in pregnancy [9,13]. Toxoplasmic chorioretinitis can be acquired congenitally or postnatally, or can be a result of a reactivation [9,12]. In immunocompromised patients, toxoplasmosis can be life-threatening and is generally a result of reactivation of chronic infection [12].

*T. gondii* infection may be associated with cardiac damage manifested by myocarditis, constrictive pericarditis, pericardial effusion, arrhythmias (atrial and ventricular), and acute heart failure [14,15,16]. According to the World Health Organization (WHO), cardiovascular diseases (CVDs) are the leading cause of death globally, killing approximately 17.9 million people each year (an estimated 32% of all deaths worldwide) [17]. However, the seroepidemiology of *T. gondii* infection in cardiovascular patients is poorly discussed in the existing literature due to apparently rare cardiovascular involvement (often asymptomatic or blurred by neurological deterioration) in toxoplasmosis [18].

Currently, two approaches are used for diagnosis of infection with *T. gondii*: (i) indirect methods (through immunological assays), and (ii) direct methods (parasite detection by microscopy and parasite DNA detection by molecular techniques) [12,19]. Indirect serological methods are widely used to evaluate the presence of specific anti-*T. gondii* antibodies (IgG, IgM, IgA) in immunocompetent individuals. Anti-*T. gondii* IgG antibodies have a lifelong persistence in humans. Serologic diagnosis is extremely useful to identify the moment of infection (based on IgG avidity test results) in pregnant women, organ donors or transplant recipients, and patients with uveitis or retinochoroiditis [5,9,12,19]. 

Recent reports from Western Romania showed a 46.09% prevalence of *T. gondii* antibodies among women of childbearing age [20], 45.9% in blood donors [6], 55.8% in pregnant women [21], 64.8% in the adult population [22], and 67.86% in psychiatric patients [23]. However, there is no data regarding the magnitude of *T. gondii* infection in cardiovascular patients and no comprehensive study was conducted to evaluate the presence of *T. gondii* antibodies in Romanian patients with cardiovascular diseases. Therefore, in the current study we aimed to evaluate the presence of specific IgG and IgM anti-*T. gondii* antibodies in both cardiovascular patients and control subjects to assess the potential relationship between cardiovascular diseases and toxoplasmosis.

## 2. Materials and Methods

### 2.1. Study Design and Study Population

A case–control study was conducted between 1 July and 31 October 2019. In the study group, 256 volunteer patients with cardiovascular diseases, admitted to the Institute of Cardiovascular Diseases in Timisoara, were enrolled in the study. The Institute of Cardiovascular Diseases is a reference healthcare institution where patients from Western Romania are evaluated and treated for cardiovascular diseases. Clinical diagnoses were established in accordance with the International Classification of Diseases 10th Revision (ICD-10) [24]. The control group consisted of 261 consecutive volunteer blood donors (with no known cardiovascular diseases) presented to the Regional Blood Transfusion Center in Timisoara. Blood donors with liver cirrhosis, chronic hepatitis, cancer, diabetes, HIV, epilepsy, schizophrenia, or anemia were excluded from blood donation according to the donation eligibility criteria set by the Romanian Ministry of Health [25]. Study and control subjects were matched by age and sex. 

Blood samples were collected by venipuncture into Serum Separation Gel & Clot Activator Activator Vacuum Tubes at study enrollment. The sera obtained after centrifugation (4000× *g* for 10 min) were moved into sterile Centrifuge Eppendorf Tubes and stored at −20 °C until tested. 

From the electronical databases of the Institute of Cardiovascular Diseases and Regional Blood Transfusion Center, subjects’ demographic data (age, sex, area of residence) were extracted using a code, without their identification. Study participants were grouped according to their age (at the time of blood collection) in 5 age groups: 19–29 years, 30–39 years, 40–49 years, 50–59 years, and 60 years and over.

### 2.2. Serologic Tests

Serologic tests were performed at the Center for Diagnosis and Study of Parasitic Diseases, Victor Babes University of Medicine and Pharmacy, Timisoara, Romania. All serum samples were tested using a Pastorex Toxo kit (Bio-Rad, Marnes-la-Coquette, France), which simultaneously detects IgG and/or IgM antibodies to *T. gondii* using latex particle agglutination. This test has shown an excellent ability to detect *T. gondii* antibodies in patients with acute and chronic toxoplasmosis [22,26,27]. Testing, quality controls, and interpretation of results were based on the manufacturer’s criteria. 

### 2.3. Statistical Analyses 

Statistical analyses were performed using MedCalc for Windows, version 19.4 (MedCalc Software, Ostend, Belgium) and the Epi Info statistical package, version 3.3.2 (Centers for Disease Control and Prevention, Atlanta, GA, USA). Data are presented as number, percentage, mean ± standard deviation (SD), and odds ratio (OR) with 95% confidence interval (CI). For comparison between *T. gondii*-positive and -negative study participants we used Mantel–Haenszel chi-square and two-tailed Fisher’s exact tests. A *p*-value of <0.05 was considered to represent statistical significance. 

### 2.4. Ethical Consideration and Informed Consent

This study was approved by Victor Babes University of Medicine and Pharmacy Timisoara Ethics Committee (No 6/2018). All study participants provided written informed consent after they were informed about the study goals and the procedures used.

## 3. Results

The 256 patients with cardiovascular diseases enrolled in the study were aged between 19 and 77 years (mean age = 47.82 ± 9.93 years), 173 (67.58%) were males, and 141 (55.08%) were residents of urban areas. In the control group, the 261 blood donors were aged between 19 years and 63 years (mean age = 46.35 ± 10.56 years), 167 (64%) were males, and 180 (68.97%) were residents of urban areas (Table 1). No significant difference in age was found between cases and controls (*p* = 0.25).

The overall seroprevalence of *T. gondii* antibodies was 64.06% (164/256) in patients with cardiovascular diseases and 52.88% (138/261) in blood donors, and tended to increase with age in both groups (Table 2).

In the study group, the seroprevalence of *T. gondii* antibodies was significantly associated with age and increased from 26.32% (5/19) in age group 19–29 years to 48.0% (12/25) (*p* = 0.21; OR: 2.58; 95%CI: 0.71–9.36) in age group 30–39 years, to 59.34% (54/91) (*p* = 0.01; OR: 4.08; 95%CI: 1.35–12.31) in age group 40–49 years, to 75.86% (88/116) (*p* = <0.001; OR: 8.80; 95%CI: 2.91–26.59) in age group 50–59 years, to 100% (5/5) (*p* = 0.005) in patients with cardiovascular diseases aged 60 years and over (Table 2). Statistical analysis performed in the control group revealed a significant association between seroprevalence of *T. gondii* antibodies and age, with increasing values of seroprevalence from 18.18% (4/22) in age group 19–29 years to 22.22% (6/27) (*p* = 1.0; OR: 1.28; 95%CI: 0.31–5.28) in age group 30–39 years, to 53.33 (48/90) (*p* = 0.003; OR: 5.14; 95%CI: 1.61–16.40) in age group 40–49 years, to 64.61% (76/118) (*p* < 0.001; OR: 8.14; 95%CI: 2.58–25.64) in age group 50–59 years, to 100% (4/4) (*p* = 0.004) in blood donors aged 60 years and over. However, no significant difference was observed between cardiovascular patients and blood donors from the same age group regarding the seroprevalence of *T. gondii* antibodies (Table 2).

When data were analyzed according to sex, a significantly higher prevalence of *T. gondii* infection was found in cardiovascular male patients (69.94%, 121/173) compared to male blood donors (55.69%, 93/167) (OR: 1.85; 95%CI: 1.18–2.89; *p* = 0.006). No significant difference was found between cardiovascular female patients (51.80%, 43/83) and female controls (47.87%, 45/94) (OR: 1.17; 95%CI: 0.64–2.11; *p* = 0.6) (Table 3).

Further analysis with stratification by area of residence revealed that *T. gondii* seroprevalence tended to be higher in cardiovascular patients residing in rural areas (73.04%, 84/115) compared to controls from rural areas (60.49%, 49/81), but with no significant difference (OR: 1.77; 95%CI: 0.96–3.25; *p* = 0.06). Likewise, no significant difference in *T. gondii* seroprevalence was found when cardiovascular patients from urban areas were compared with blood donors from urban areas (56.74%, 80/141 vs. 49.44%, 89/180; OR: 1.34; 95% CI: 0.86–2.08, *p* = 0.21) (Table 3).

The seroprevalences of anti-*T. gondii* antibodies in patients with cardiovascular diseases according to their diagnostics are presented in Table 4. When compared to the control group, a significantly higher prevalence of *T. gondii* antibodies was found among patients with hypertension (82.35%, 14/17; OR: 4.15; 95%CI: 1.16–14.81; *p* = 0.01) and unstable angina (67.56%, 50/74; OR: 1.85; 95%CI: 1.07–3.19; *p* = 0.02) (Table 4).

## 4. Discussion

Toxoplasmosis, one of the most common infections in humans, has a worldwide distribution. Prevalence varies widely from one country to another (from less than 10% to over 90%), and often between different communities in the same region [5,9,28]. High prevalence is reported in Latin America, areas of Central and Eastern Europe, the Middle East, areas of Africa and South-East Asia [19]. Toxoplasmosis prevalence is influenced by several factors: (i) the number and presence of cats; (ii) socioeconomic conditions (with higher prevalence in underdeveloped countries compared to developed ones); (iii) environmental conditions (oocysts lose their virulence when frozen or dried, and therefore higher prevalence is observed in areas of lower altitudes, with warm and humid climate); (iv) cultural habits; (v) diet and cooking habits; (vi) hygiene; (vii) host susceptibility [9,19,28].

In human toxoplasmosis, the most frequently affected organs are the brain and the heart, with more damage in the case of immune deficiency through reactivation of the latent parasite [15]. Moreover, patients with history of cardiovascular diseases may be more predisposed to chronic infection with *T. gondii* [18]. In toxoplasmosis, cardiovascular involvement is unusual, myocarditis being the most commonly described. Despite significant histopathological changes that occur at the site of cardiac lesions caused by *T. gondii*, clinical manifestations of toxoplasmic myocarditis vary widely from asymptomatic cases or patients with nonspecific symptoms to a self-limiting disease or cases with fulminant evolution (patients with severe left ventricular dysfunction requiring hemodynamic support). Arrhythmias, cardiomyopathy, heart failure and cardiac arrest are among the most described complications [18,29,30].

The heart is the second most affected organ (after the brain) in patients with acquired immunodeficiency syndrome (AIDS). Approximately 12–22% of AIDS patients had evidence of cardiac involvement confirmed at autopsy, due to the absence of cardiac clinical signs. The introduction of highly antiretroviral therapy in the medical management of patients with AIDS led to a decrease in prevalence of cardiac toxoplasmosis in these patients to less than 10% [31].

In transplant recipients, *T. gondii* infection may be a result of a primary infection (transmission of the parasite from a seropositive donor to a seronegative recipient) or a reactivation of a latent infection of the recipient [10,32]. In the case of solid organ transplantation, the risk of *T. gondii* transmission is potentially high compared to allogeneic hematopoietic stem cell transplantation, when the risk is more theoretical than real. Cysts of *T. gondii* rarely form in the liver, pancreas, kidney, or intestine, and consequently the risk of transmission in the case of these transplanted organs is very low [10]. In patients undergoing heart transplantation, toxoplasmosis is the most commonly reported parasitic disease, with a higher risk of developing *T. gondii*-induced myocarditis [18,31,32]. In these patients, reactivation of latent toxoplasmosis can be explained by the high predisposition of *T. gondii* to persist as tissue cysts in striated muscle [33,34]. In the myocardium, *T. gondii* can trigger a local inflammatory response: parasite proliferation leads to myocyte ruptures followed by a fugacious exudative or neutrophilic component which rapidly becomes mononuclear [32,35]. The histopathological examination can highlight myonecrosis, edema, and inflammatory cell infiltrate (with macrophages, plasma cells, lymphocytes, and eosinophils) [35]. Myocarditis is frequently associated with involvement of other organs, most frequently the brain and the lungs. Disseminated toxoplasmosis with associated myocarditis can be fatal in the case of no prior *T. gondii* prophylaxis [31,35]. *T. gondii* tissue cysts are often present in non-inflamed myocardium, with no myocyte rupture and the parasite being hidden from the immune system [32].

Limited data are currently available on the seroprevalence of *T. gondii* in patients with cardiovascular diseases, and most of them are case reports of immunocompromised individuals (infected with HIV or transplant recipients) [18].

This is the first study that assessed the seroprevalence of *T. gondii* in Romanian cardiovascular patients, investigating at the same time the possible relationship between *T. gondii* and cardiovascular diseases.

The 64.06% seroprevalence of *T. gondii* antibodies among Romanian patients diagnosed with cardiovascular diseases was similar to the 63.73% seroprevalence reported in Iran [36], 63.1% in Egypt [37], and higher than the 13.8% prevalence found in Mexico [14].

In their study conducted in Urmia City, Iran, Khademvatan and colleagues obtained a 63.73% seroprevalence of anti-*T. gondii* IgG antibodies, significantly higher in patients suffering from heart disease compared to the 37.64% seroprevalence found in controls (*p* < 0.01). These findings suggest that *T. gondii* antibodies may be more frequently detected in patients with heart diseases. With advancing age, there is a reduction in muscle contractility and a greater susceptibility to high blood pressure and diabetes. Moreover, due to *T. gondii* tissue cysts located in heart muscles, *T. gondii*-infected individuals can be more prone to cardiovascular problems compared to those uninfected [36].

Seroprevalence of *T. gondii* antibodies increased with age in our study, in both case and control groups, and this is consistent with the result published by Alvarado-Esquivel et al. [14]. Longer exposure of older individuals to sources of infection may explain the result [38]. Therefore, to properly evaluate the potential association between *T. gondii* infection and patients with cardiovascular diseases, cases and controls were matched by age.

Cardiovascular male patients were significantly more infected with *T. gondii* compared to male blood donors in our study. Similar results were obtained by Khademvatan and colleagues: the seroprevalence of anti-*T. gondii* IgG antibodies was significantly higher in male patients diagnosed with chronic heart diseases compared to healthy volunteers [36].

In Romanian patients diagnosed with acute myocardial infarction, prevalence of *T. gondii* infection tended to be higher (65.59%) compared to blood donors (52.88%), with no significant difference. In Iran, two studies were conducted to evaluate the prevalence of *T. gondii* infection and its association with myocardial infarction. Gohari et al. showed that the seroprevalence of *T. gondii* IgG antibodies was significantly higher in patients suffering from myocardial infarction (61.6%) compared to controls (24.4%) [39]. Hamidinejat et al. found a significantly higher seroprevalence of *T. gondii* IgG and IgM antibodies in patients with acute myocardial infarction (66.6% and 22.9%, respectively) compared to healthy volunteers (29.2% and 4.2%, respectively) [40]. During the acute phase of infection, *T. gondii* tachyzoites replicate and cause necrosis of the myocytes. Therefore, in *T. gondii*-infected patients with acute myocardial infarction, acute myocarditis should be considered as a possible diagnosis due to the fact that its clinical signs may hide under the mask of acute myocardial infarction [40].

In the present study, the seroprevalence of *T. gondii* was 71.42% in patients with atherosclerotic heart disease, higher than the 52.88% seroprevalence in the control group, but with no significant difference. Alhusseiny et al. revealed, in their study conducted in Egypt, that the prevalence of anti-*T. gondii* antibodies was 63.1% in atherosclerotic patients compared to 42.6% in non-atherosclerotic individuals [37]. In latent *T. gondii* infections, a periodic rupture of tissue cysts (with bradyzoites set free) occurs spontaneously in intermediate hosts [41]. A pathophysiological process may be triggered, followed by vascular injury and inflammation. In immunocompetent individuals, released bradyzoites are cleared by the immune system [42,43]. In response to inflammation, vascular endothelial cells will release into circulation adhesion molecules (soluble intercellular adhesion molecule 1 and soluble vascular cell adhesion molecule 1), mediating leukocyte adherence to the vascular endothelium and transmigration. Development of atherosclerosis was previously linked to chronically elevated levels of these adhesion molecules [43,44].

Our findings showed a significantly higher seroprevalence of *T. gondii* antibodies in patients with hypertension (82.35%) compared to blood donors. Similarly, Babekir et al. revealed that *T. gondii* IgG antibody-positive individuals had significantly higher systolic blood pressure compared to negative ones (*p* = 0.0022) [15]. *T. gondii* causes cardiovascular dysfunction through a complex mechanism. The increase in oxidative stress and inflammation has an adverse cardiovascular outcome. IFN regulatory factors (IRFs), the nuclear factor κB (NF-κB), and mitogen-activated protein kinases (MAPKs) play critical roles in the induction of tumor necrosis factor α (TNFα), interferons (IFN) type I and type II, and interleukin (IL) 12 [8,15,45]. By increasing the production of nitric oxide (NO) and reactive oxygen species (ROS), *T. gondii* increase vasoconstriction and oxidative stress in tissues, followed by atherosclerotic plaque production, lipid accumulation, foam cell formation, and inflammation. Endothelial injury, platelet activation, and vascular modeling are listed among adverse cardiovascular outcomes driven by oxidative stress [15,28,46].

Results of our survey indicate a significantly higher seroprevalence of *T. gondii* antibodies in patients with unstable angina compared to controls. Vasospasm, elevated blood pressure, intraluminal thrombosis, and intraluminal plaque formation can block the blood flow to the myocardium, resulting in unstable angina [47]. As described above, *T. gondii* is able, through a complex mechanism, to model blood vessels, provoke vasoconstriction, activate platelets, and induce atherosclerotic plaque production [8,15,28,43,44,45,46].

No significant difference in *T. gondii* seroprevalence was found between patients diagnosed with congestive heart failure and healthy blood donors in our study (*p* = 0.45). In contrast, Yazar and colleagues found that *T. gondii* IgG seropositivity was significantly higher in patients with chronic heart failure than in healthy volunteers (*p* < 0.05) in a study conducted in Turkey [48].

The present study is subject to certain limitations. Our case–control survey analyzed data collected at one specific point in time and cannot prove causality, since temporality of association is a strong criterion [15]. However, our results suggest that patients with hypertension and unstable angina may be more frequently infected with *T. gondii* compared to healthy individuals. Sample size may be listed as another limitation. Due to the limited number of patients included in each type of cardiovascular disease, it was difficult to properly evaluate *T. gondii* seroprevalence in each group and investigate the influence of the parasite on cardiac function. Further studies, with higher number of participants will be ideal in confirming our results. Our sampling less represented females and this may be another limitation. It is well known that there are disparities in the susceptibility and development of cardiovascular diseases between females and males, usually with lower incidence in women than in men. This phenomenon is caused by innate genes, certain differences in sex hormones, in cardiac structure and function, environmental influences and socio-psychological characters [49,50]. Another limitation is the small number of study participant aged over 60 years enrolled in our study. There is a significant correlation between cardiovascular diseases and age: the risk of cardiovascular disease increases over time [49]. On the other side, blood donors are generally healthy individuals, with a limited range of age [6]. Therefore, to avoid any bias due to age difference, we matched cases and controls by age.

## 5. Conclusions

The present study brings new epidemiological information on the prevalence of *T. gondii* in patients with cardiovascular diseases from Western Romania. Our results indicate a significantly higher *T. gondii* seroprevalence in patients diagnosed with hypertension and unstable angina, suggesting that individuals with these diagnoses may be more frequently infected with *T. gondii*. This study may be a valuable starting point for further research to better evaluate the impact of *T. gondii* exposure on cardiovascular diseases.

## Figures and Tables

**Table 1:** Demographic characteristics of the study group (patients with cardiovascular diseases) and the control group (blood donors) included in the study.

Characteristics	Study Group (*n* = 256)	Control Group (*n* = 261)
**Age** (Mean ± SD)	47.98 ± 9.98	46.39 ± 10.73
**Sex**		
Male	173 (67.58%)	167 (64%)
Female	83 (32.42%)	94 (36%)
**Area of Residence**		
Urban	141 (55.08%)	180 (68.97%)
Rural	115 (44.92)	81 (31.03%)
**Age Groups** (years)		
19–29	19 (7.42%)	22 (8.43%)
30–39	25 (9.77%)	27 (10.35%)
40–49	91 (35.55%)	90 (34.48%)
50–59	116 (43.31%)	118(45.21%)
≥60	5 (1.95%)	4 (1.53%)

**Table 2:** Seroprevalence of *Toxoplasma gondii* antibodies in the study group (patients with cardiovascular diseases) and the control group (blood donors), according to age group.

	Study Group		Control Group		OR (95%CI)	*p*-Value
	No. Positive (%)	No. Negative (%)	No. Positive (%)	No. Negative (%)		
**Age group** (years)						
19–29	5 (26.32%)	14 (73.68%)	4 (18.18%)	18 (81.82%)	1.60 (0.36–7.12)	0.70
30–39	12 (48.0%)	13 (52.0%)	6 (22.22%)	21 (77.78%)	3.23 (0.97–10.72)	0.07
40–49	54 (59.34%)	37 (40.66%)	48 (53.33%)	42 (46.67%)	1.27 (0.70–2.30)	0.45
50–59	88 (75.86%)	28 (24.14%)	76 (64.41%)	42 (35.59%)	1.73 (0.98–3.06)	0.06
≥60	5 (100.0)	0 (0.0%)	4 (100%)	0 (0.0%)	NA	0.1

NA, not applicable.

**Table 3:** Seroprevalence of *Toxoplasma. gondii* antibodies in the study group (patients with cardiovascular diseases) and the control group (blood donors), according to sex and area of residence.

	Study Group		Control Group		OR (95%CI)	*p*-Value
	No. Positive (%)	No. Negative (%)	No. Positive (%)	No. Negative (%)		
**Sex**						
Male	121 (69.94%)	52 (30.06%)	93 (55.69%)	74 (44.31%)	1.85 (1.18–2.89)	0.006
Female	43 (51.80%)	40 (48.20%)	45 (47.87%)	49 (52.13%)	1.17 (0.64–2.11)	0.60
**Area of Residence**						
Urban	80 (56.74%)	61 (43.26%)	89 (49.44%)	91 (50.56%)	1.34 (0.86–2.08)	0.21
Rural	84 (73.04%)	31 (29.96%)	49 (60.49%)	32 (39.50%)	1.77 (0.96–3.25)	0.06

**Table 4:** Seroprevalence of *Toxoplasma gondii* antibodies in patients with cardiovascular diseases from Western Romania, according to diagnosis.

Diagnosis	ICD-10 Diagnosis	No. Tested	No. Tested Positive (%)	OR (95%CI)	*p*-Value *
Acute myocardial infarction	I21	47	31 (65.95%)	1.72 (0.90–3.30)	0.09
Aortic aneurysm	I71	5	2 (0.4%)	0.59 (0.09–3.61)	0.56
Arrhythmia	I49.9	27	13 (48.14%)	0.82 (0.37–1.82)	0.63
Atherosclerotic heart disease	I25.1	28	20 (71.42%)	2.22 (0.94–5.24)	0.06
Congestive heart failure	I50	16	10 (62.5%)	1.48 (0.52–4.20)	0.45
Dilated cardiomyopathy	I42.0	7	6 (85.71%)	5.34 (0.63–45.04)	0.08
Hypertension	I10	17	14 (82.35%)	4.15 (1.16–14.81)	0.01
Myopericarditis	I30-I40	3	1 (33.33%)	0.44 (0.03–4.97)	0.50
Pulmonary oedema/ Pulmonary embolism	J81.1-I26	3	2 (66.66%)	1.78 (0.15–19.90)	0.63
Unstable angina	I20.0	74	50 (67.56%)	1.85 (1.07–3.19)	0.02
Valvular heart disease	I05-I09	29	15 (51.72%)	0.95 (0.44–2.05)	0.90

* The seroprevalence of *Toxoplasma gondii* antibodies was compared with the 52.88% seroprevalence in the control group.

## Data Availability

Data are available upon request.
